# Pregnancy intendedness and the association with physical, sexual and emotional abuse – a European multi-country cross-sectional study

**DOI:** 10.1186/s12884-015-0558-4

**Published:** 2015-05-26

**Authors:** Mirjam Lukasse, Made Laanpere, Helle Karro, Hildur Kristjansdottir, Anne-Mette Schroll, An-Sofie Van Parys, Anne-Marie Wangel, Berit Schei, On behalf of the Bidens study group

**Affiliations:** Institutt for Health, Nutrition and Management, Faculty of Health Sciences, Oslo and Akershus University College of Applied Sciences, Oslo, Norway; Department of Obstetrics and Gynaecology,, University of Tartu, Tartu, Estonia; Tartu University Hospital Women’s Clinic, Tartu, Estonia; Department of Midwifery, Faculty of Nursing, University of Iceland, Reykjavik, Iceland; Department of Ultrasound, Rigshospitalet, Copenhagen, Denmark; Department of Obstetrics and Gynaecology, Ghent University Hospital, Ghent, Belgium; Faculty of Health and Society, Malmö University, Malmö, Sweden; Department of Public Health and General Practice, Norwegian University of Science and Technology, Trondheim, Norway; Department of Obstetrics and Gynaecology, St. Olav’s University Hospital, Trondheim, Norway

**Keywords:** Unintended pregnancy, Sexual abuse, Physical abuse, Emotional abuse, Pregnancy intention

## Abstract

**Background:**

Unintended pregnancies are common and when not resulting in a termination of pregnancy may lead to unintended childbirth. Unintended pregnancies are associated with increased health risks, also for women for whom pregnancy continues to childbirth. Our objective was to present the prevalence of unintended pregnancy in six European countries among pregnant women attending routine antenatal care, and to investigate the association with a history of physical, sexual and emotional abuse.

**Methods:**

A prospective cross-sectional study, of 7102 pregnant women who filled out a questionnaire during pregnancy as part of a multi-country cohort study (Bidens) with the participating countries: Belgium, Iceland, Denmark, Estonia, Norway and Sweden. A validated instrument, the Norvold Abuse Questionnaire (NorAq) consisting of 10 descriptive questions measured abuse. Pregnancy intendedness was assessed using a single question asking women if this pregnancy was planned. Cross-tabulation, Chi-square tests and binary logistic regression analysis were used.

**Results:**

Approximately one-fifth (19.2 %) of all women reported their current pregnancy to be unintended. Women with an unintended pregnancy were significantly younger, had less education, suffered economic hardship, had a different ethnic background from the regional majority and more frequently were not living with their partner. The prevalence of an unintended pregnancy among women reporting any lifetime abuse was 24.5 %, and 38.5 % among women reporting recent abuse. Women with a history of any lifetime abuse had significantly higher odds of unintended pregnancy, also after adjusting for confounding factors, AOR for any lifetime abuse 1.41 (95 % CI 1.23–1.60) and for recent abuse AOR 2.03 (95 % CI 1.54–2.68).

**Conclusion:**

Women who have experienced any lifetime abuse are significantly more likely to have an unintended pregnancy. This is particularly true for women reporting recent abuse, suggesting that women living in a violent relationship have less control over their fertility.

**Electronic supplementary material:**

The online version of this article (doi:10.1186/s12884-015-0558-4) contains supplementary material, which is available to authorized users.

## Background

Unintended pregnancy and unintended childbirth can have serious health, economic, and social consequences for women and their families. Unintended childbearing is associated with antenatal depression, continued risk behavior including alcohol consumption and smoking, failure to adapt health-improving behaviors like taking folic acid and failure to initiate early antenatal care [1–5]. Unintended childbirth additionally increases the risk for low birth weight and preterm birth, postnatal depression, and negatively affects breastfeeding and bonding [6–9].

Studies suggest that 9–65 % of births worldwide are the result of a pregnancy that was not planned [10–15]. The great variation in this prevalence is due to differences in sampling and methodology as well as differences between cultures and countries. An unintended pregnancy may be unwanted, mistimed or unexpected [15–18]. Studies suggest that the negative impact of unintended pregnancies is greater for unwanted than for mistimed or unexpected pregnancies [7, 16].

Unintended pregnancy is more common in younger women, single women, women not cohabiting with their partner, and women with more children [10, 19]. Studies report that race, ethnicity and recent immigration influence the prevalence of unintended pregnancy and birth [19, 20]. These studies suggest this may be due to racial differences in women’s willingness to terminate unwanted pregnancies and ethnic and religious issues regarding the use of family planning and co-habiting. Furthermore, studies suggest that women experiencing intimate partner violence (IPV) are at greater risk of unintended pregnancy [15, 21]. The proposed mechanisms for this are, forced sex, fear of negotiating contraceptive use, birth control sabotage, and partner interference with access to healthcare [22, 23]. Unintended pregnancy does not necessarily lead to unintended birth, but more frequently to termination of pregnancy [24]. This is why much of the research on the association between violence against women and unintended pregnancy has been performed by those working in the field of contraception and gynecology as opposed to obstetrics [23, 25, 26]. Few studies from high-economic countries have described the association between a history of physical, sexual or emotional abuse and the occurrence of unintended pregnancy among women planning to give birth [21]. Those that do, present crude associations without adjusting for obvious confounding factors, such as age, education, marital- and economic status [14, 16]. The first objective of our study was to present the prevalence of unintended pregnancies among pregnant women intending to give birth in 6 European countries. The second objective of was to investigate the association between unintended pregnancy and a history of physical, emotional or sexual abuse by any/unknown perpetrator(s).

## Methods

This prospective cross-sectional study uses data collected during pregnancy as part of the Bidens cohort study, a six-country (Belgium, Iceland, Denmark, Estonia, Norway, and Sweden) study of women attending routine antenatal care, between March 2008 and August 2010 [27].

The main aim of the Bidens cohort study was to assess the association between a history of abuse and mode of delivery and this determined the population size [27]. A total of 7200 pregnant women who consented, subsequently completed a questionnaire and allowed for the extraction of specified data on delivery from their medical notes. The 68 -item questionnaire included several validated instruments measuring fear of childbirth, abuse and depression. Where a previously translated version of the instruments were available, these were used. Otherwise, the questionnaire was translated into the required languages by a native speaker (Flemish, Icelandic, Danish, Estonian, Russian, Norwegian and Swedish) and then translated back again into the source language. The original and back-translated versions were used to determine the final version.

Due to country specific organization as well as requirements of local ethic committees, minor variations in the recruitment procedure occurred. In Belgium, women were approached by the midwife or secretary when attending antenatal care. Consenting women were asked to complete the questionnaire in a separate room. In Iceland women were recruited when attending routine ultrasound and returned completed forms by mail. In Denmark women were given information about the study when attending early routine ultrasound screening and were mailed the questionnaire later. They returned the questionnaire by mail or when attending their next ultrasound examination. In Estonia women were invited to participate while visiting for an antenatal consultation. After completing the questionnaire it was left in a mailbox at the clinic. In Norway women, after attending routine ultrasound, received the questionnaire by mail and returned it by mail. Non-responders were sent one reminder. In Sweden, the questionnaire was administered to women when attending routine glucose tolerance tests and filled out during the two hours gap between the blood samplings. Belgium and Sweden were not permitted to record non-participation. The estimated response rate varied between 50 % in Norway to 90 % in Estonia.

All women required sufficient language skills to fill out the questionnaire. In Estonia women could choose to complete an Estonian or Russian language questionnaire. In Belgium, Iceland and Denmark women less than 18 years of age were excluded. In Denmark, only women from the local geographical area were invited. In Belgium, women who were not able to be separated from their accompanying person were not recruited. In Iceland, Denmark and Norway, women with major fetal pathologies were excluded from the study.

### Ethics

The study was conducted in accordance with the ethical guidelines developed by the World Health Organization (WHO) [28]. The information letter instructed women to complete the form in a place where they could be undisturbed and included telephone numbers and e-mail addresses to contact if needed. Additionally, in Belgium, Estonia and Sweden, participants had the opportunity to complete the questionnaires at the clinic, and measures were installed to ensure accompanying persons were not with them. Data was anonymized before analysis. Formal approvals of local ethical committees were obtained in each country, as listed below.

Belgium: The Ethical Committee of Ghent University acted as the central ethical committee for the study; U(Z) Gent, 22012008/B67020072813, date of approval: 1st February 2008, Waregem hospital date added: 21st October 2008. Iceland: The scientific board approved the study (24.06.2008-VSN-b2008030024/03-15) according to Icelandic regulations, date: 24th June 2008. In Denmark, even though ethical approval for non-invasive studies is not required, the study was presented to the Research Ethics Committee of the Capital Region, who found no objections to the study (H-A-2008-002), date: 11th February 2008. Permission was obtained from the Danish Data Protection Agency (J.nr. 2007-41-1663). In Estonia, ethical permission was given by the Ethics Review Committee on Human Research of the University of Tartu, Estonia; 190/M-29, 192/-22, 196/X-2, date: 17th December 2007, East-Tallinn Central Hospital added: 19th January 2009, Russian language and prolonged period added: 22nd February 2010, East-Viru Central Hospital added: 26th April 2010. In Norway, the Regional Committee for Medical Research Ethics in North approved the study (72/2006), date: 29th August 2007; and the Data Inspectorate (NSD) (15214/3/) also approved the study, date: 19th December 2007. In Sweden, the study was approved by the Regional Ethical Committee in Stockholm (2006/354-31/1), date: 14th June 2006.

### Definition of variables

**Fig. 1:**
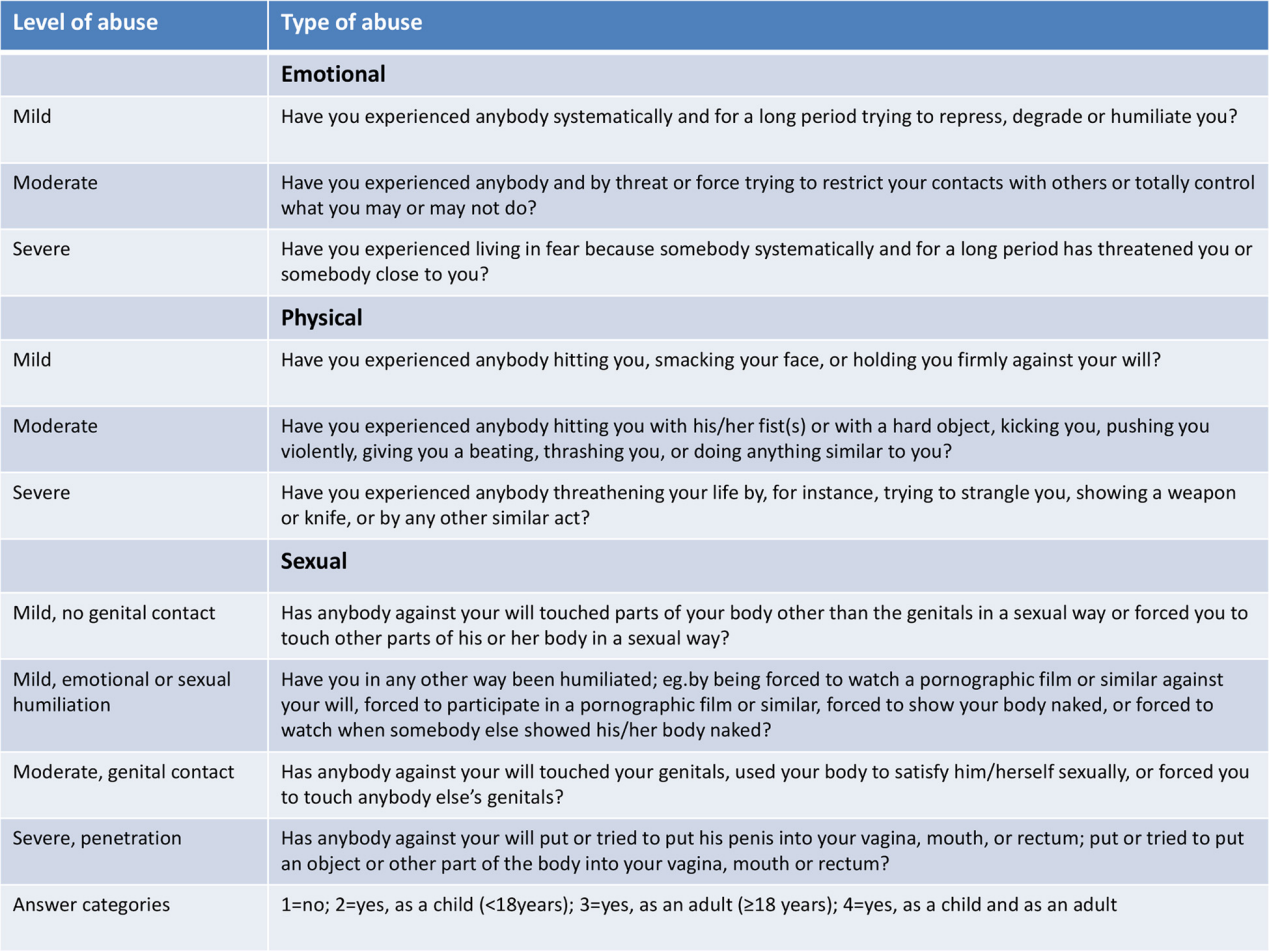
The Norvold Abuse questions (NorAq)

Whether a pregnancy was intended or not was assessed using a single question which in 5 of the 6 countries was worded “Was this pregnancy planned?” with a “yes” or “no” answering option. In Sweden it was considered culturally appropriate to pose the question as “Was this pregnancy unplanned?” with the same response options. The responses were harmonized through coding. The questionnaire included questions on socio-economic background, general and mental health, and obstetric history. The questions on abuse were taken from the Norvold Abuse Questionnaire (NorAQ), which was developed in a Nordic multi-center study among gynecological patients [29]. This validated instrument includes 10 descriptive questions measuring emotional, physical, and sexual abuse at increasing levels of severity, i.e. mild, moderate and severe (Fig. 1) [30]. For each type and level of abuse the answer categories were “no”, “yes as a child”, “yes as an adult”, or “yes both as a child and as an adult”. The responses were classified according to the most severe level reported and categorized as either adult or childhood abuse. Women were defined as having experienced any abuse/any lifetime abuse if they answered yes to at least one of the questions of sexual, emotional and physical abuse [29]. The question measuring mild physical abuse has shown low specificity in the validation study and was therefore excluded [30].

In addition, women were asked if they had experienced the abuse during the past 12 months, which in our study was termed recent abuse. Parity was derived from a question asking women how many children they had given birth to. Women reported their education by checking one of four predefined categories shown in Table 1. Economic hardship was investigated by asking women how easy it would be for them to pay a large bill (3070 Euro or 4230 US $) within a week, adjusted by countries’ consumer price index (CPI). The answering option “very difficult” was defined as experiencing economic hardship. Women were asked how many weeks pregnant they were when filling out the form, which was categorized as shown in the Table 1. Women whose mother tongue was the national language were considered as ethnically belonging. As we recruited in Flanders in Belgium this was Flemish, and in Estonia this was Estonian.

**Table 1:** Participant characteristics by pregnancy intention among women in the Bidens study, N = 7102

	Unintended pregnancy		Intended pregnancy		P-value
	n = 1367		n = 5735		
Age					
<25	334	(24.4)	575	(10.0)	<0.001
25–30	462	(33.8)	2433	(42.4)	
31–35	374	(27.4)	1919	(33.5)	
>35	185	(13.5)	774	(13.5)	
Missing	12	(0.9)	34	(0.6)	
Education					
<10 years	111	(8.1)	126	(2.2)	<0.001
10–13 years	487	(35.6)	1324	(23.1)	
>13 years	742	(54.3)	4213	(73.5)	
Missing	27	(2.0)	72	(1.3)	
Civil status					
Married/cohabiting	1150	(84.1)	5570	(97.1)	
Other	188	(13.8)	106	(1.9)	<0.001
Missing	29	(2.1)	59	(1.0)	
Occupation					
Unemployed	81	(5.9)	138	(2.4)	<0.001
Student	197	(14.4)	433	(7.6)	
Employed or freelancer	886	(64.8)	4536	(79.1)	
Sick leave or rehabilitation	36	(2.6)	54	(0.9)	
Pregnancy leave	87	(6.4)	403	(7.0)	
Housewife	63	(4.6)	125	(2.2)	
Missing	17	(1.2)	46	(0.8)	
Ethnicity					
Mother tongue national language	1171	(85.7)	5178	(90.3)	
Mother tongue not national language^α^	196	(14.3)	557	(9.7)	<0.001
Economic hardship					
No	795	(58.5)	4327	(75.5)	<0.001
Yes	534	(39.1)	1279	(22.3)	
Missing	38	(2.4)	129	(2.2)	
Gestational age					
<20	175	(12.8)	933	(16.3)	<0.001
20–24	520	(38.0)	2177	(38.0)	
25–30	529	(38.7)	1958	(34.1)	
>30	129	(9.4)	636	(11.1)	
Missing	14	(1.0)	31	(0.5)	

^α^includes women who filled out Russian language form in Estonia

### Statistical analyses

Cross-tabulation and Pearson’s Chi square test were performed to compare the prevalence of pregnancy intention between countries and by selected socio-economic and obstetric factors. Level of significance was set at p < 0.05, two-sided. The association between pregnancy intention and the different types of abuse was assessed by calculating Crude and Adjusted Odds Ratios (OR) with 95 % Confidence Intervals (CI) using binary logistic regression analysis. Based on the literature we entered the following a priori selected covariates in the first model: age, education, civil status, occupation, ethnicity, economic hardship and gestational age. Neither occupation nor ethnicity influenced the adjusted OR and they were therefore excluded from the final models presented in the paper. Comparison group consisted of women with no sexual, physical or emotional abuse. Analyses were performed in PASW Statistics (version 22.0).

## Results

From the 7200 women who filled out the questionnaires we excluded 39 women for whom we lacked information pregnancy intendedness and 59 women who failed to answer any of the questions for either emotional, sexual or physical abuse, resulting in a sample of 7102 women. Approximately one-fifth (19.2 %) of all women reported their current pregnancy to be unintended (Table 2). There were significant differences between the participating countries. The lowest prevalence was found in Belgium (10 %), while Iceland had the highest prevalence (26 %) (Table 2). These differences remained significant after adjusting for age, civil status, education, gestational age and economic hardship (Additional file 1: Table S1).

**Table 2:** Prevalence of unintended pregnancy in 6 participating countries among women in the Bidens study, N = 7102

	Belgium	Iceland	Denmark	Estonia	Norway	Sweden	Total
	n = 851	n = 598	n = 1276	n = 963	n = 2403	n = 1012	n = 7102
	n(%)	n(%)	n(%)	n(%)	n(%)	n(%)	n(%)
Unintended pregnancy	85 (10.0)	156 (26.1)	165 (12.9)	220 (22.8)	506 (21.1)	235 (23.2)	1367 (19.2)

Chi-square P-value <0.001 for differences between the countries

Unintended pregnancy was significantly more common among women less than 25 years of age, those who had less than 13 years of education, not living with their partner, and students or women not working outside their home (Table 1). Women whose mother tongue was different form the local language at the study site were significantly more likely to report having an unintended pregnancy (Table 1).

The prevalence of an unintended pregnancy among women not reporting any abuse was 15.8 % compared to 24.5 % among those reporting any lifetime abuse (Table 3). While the prevalence of unintended pregnancy among women reporting recent sexual abuse was 51.7 % (Table 3). Women with a history of abuse had significantly higher odds of unintended pregnancy, also after adjusting for confounding factors. This was particularly true for recent abuse (Table 4).

**Table 3:** Prevalence of unintended pregnancy by history of abuse (*row percent*), the Bidens study, N = 7102

Categories of abuse	Total	Unintended pregnancy		Intended pregnancy	
No abuse	4270	674	(15.8)	3596	(84.2)
Any lifetime abuse	2832	693	(24.5)	2139	(75.5)
Any abuse <18	1672	442	(26.4)	1230	(73.6)
Any abuse ≥18	1752	457	(26.1)	1295	(73.9)
Recent abuse	309	118	(38.2)	191	(61.8)
Emotional abuse	1344	379	(28.2)	965	(71.8)
Emotional abuse <18	870	247	(28.4)	623	(71.6)
Emotional abuse ≥18	727	223	(30.7)	504	(69.3)
Recent emotional abuse	186	77	(41.4)	109	(58.6)
Physical abuse	1753	470	(26.8)	1283	(73.2)
Physical abuse <18	722	212	(29.4)	510	(70.6)
Physical abuse ≥18	1233	334	(27.1)	899	(72.9)
Recent physical abuse	142	55	(38.7)	87	(61.3)
Sexual abuse	1116	313	(28.0)	803	(72.0)
Sexual abuse <18	780	221	(28.3)	559	(71.7)
Sexual abuse ≥18	447	129	(28.9)	318	(71.1)
Recent sexual abuse	29	15	(51.7)	14	(48.3)

**Table 4:** Crude and adjusted OR for unintended pregnancy by history of abuse, the Bidens study, N = 7102

	Crude OR	Adjusted OR^§^
No abuse	Ref	Ref
Any lifetime abuse	1.76 (1.56–1.98)	1.41 (1.23–1.60)
Any abuse <18	1.92 (1.67–2.20)	1.49 (1.28–1.73)
Any abuse ≥18	1.88 (1.65–2.15)	1.50 (1.29–1.73)
Recent abuse	3.30 (2.58–4.21)	2.03 (1.54–2.68)
Emotional abuse	2.10 (1.81–2.42)	1.58 (1.35–1.85)
Emotional abuse <18	2.12 (1.79–2.50)	1.55 (1.28–1.86)
Emotional abuse ≥18	2.36 (1.98–2.82)	1.70 (1.39–2.07)
Recent emotional abuse	3.77 (2.78–5.01)	2.36 (1.67–3.32)
Physical abuse	1.95 (1.71–2.23)	1.45 (1.26–1.69)
Physical abuse <18	2.22 (1.85–2.65)	1.55 (1.27–1.89)
Physical abuse ≥18	1.98 (1.71–2.30	1.48 (1.25–1.75)
Recent physical abuse	3.37 (2.38–4.77)	1.95 (1.31–2.90)
Sexual abuse	2.08 (1.78–2.43)	1.67 (1.41–1.97)
Sexual abuse <18	2.11 (1.77–2.52)	1.66 (1.37–20.2)
Sexual abuse ≥18	2.16 (1.74–2.70)	1.73 (1.36–2.20)
Recent sexual abuse	5.72 (2.75–11.90)	2.64 (1.17–5.98)

Comparison group is women not reporting any abuse

^§^adjusted for age, education, civil status, economic hardship and gestational age

We did not observe a dose–response effect between unintended pregnancy and the categories mild, moderate and severe abuse for any of the types of abuse (Additional file 2: Table S2). When analysed by participating country, the results all pointed in the same direction. However, the adjusted association between unintended pregnancy and any lifetime abuse was only significant for Norway and Sweden (Additional file 3: Figure S1).

## Discussion

Among pregnant women attending routine antenatal care in 6 European countries, one in five women had not intended to become pregnant. Women who have experienced abuse are more likely to report their current pregnancy to be unintended. The association was significant for all the types of abuse, also after adjusting for confounding factors, and strongest for women reporting recent abuse, who were twice as likely to have an unintended pregnancy.

In our study, we assessed pregnancy intendedness using a single question, asking women if their pregnancy was “planned”. In Swedish the questions was the opposite, asking women if the pregnancy was “oplanerad” which directly translated means “unplanned” i.e. not planned. Our analyses suggest this was the appropriate way of culturally adapting the question, capturing the same concept. It may be a limitation of our study that we assessed pregnancy intention with a single question, as the concept is a complex one. American surveys have traditionally used sets of question sequences yielding prevalence estimates of a pregnancy being intended (i.e. wanted at the time the pregnancy occurred), mistimed (wanted, but not at the time it occurred) or unwanted (not wanted at any time) [31].

A qualitative study by Barrett and Wellings found that there is a vast variability in women’s understanding of “planned” and “unplanned” [32]. Still, women in this qualitative study thought the term “intended” was interchangeable with the term “planned” and they generally preferred the terms “planned” and “unplanned” while positively disliking “unwanted” [32]. The British women in Barret and Welling’s study applying the term “planned” included not only the intention to become pregnant and stopping contraceptives, but also partner agreement and the right time in their life [32]. In contrast, “unplanned” included not intending to get pregnant, failure to use contraception, failure of contraception, and accident/mistake [32]. As a result of their qualitative study, Barrett and Welling’s suggested using more than one question to assess the planning status of a pregnancy in quantitative studies and together with Smith they developed an instrument consisting of 6 questions which they stated, captures a more nuanced picture of intention [18]. However, when their instrument was used in a recent study the 6 questions were used as a measuring scale resulting in a score which then translated into two categories, unplanned and planned pregnancy [17]. The same categories we used in our study. We conclude that even though we only used a single question we very likely captured the core concept. It needs to be emphasized that women may be happy with the pregnancy even though it was not intended [33, 34].

While previous studies have shown an association between unintended pregnancy and physical and sexual intimate partner violence (IPV) our study adds the association between emotional abuse and unintended pregnancy [11, 21]. Although not shown explicitly before, emotional abuse certainly played a role in the results of the previous studies on IPV. It is through threat, degradation, humiliation and restriction of freedom a woman can lose control over her own life and decisions on fertility. A substantial proportion of women who suffer from intimate partner physical and sexual violence also are victims of emotional violence [35, 36]. Recent abuse in our study occurred within the past 12 months of filling out the questionnaire and was most strongly associated with an unintended pregnancy. Women were on average six months pregnant at the time of responding to the questionnaire. We did not inquire about the perpetrator of the abuse in our study. However, based on the literature, it is likely that the perpetrator of recent abuse is the current partner and/or father of the unborn baby [35, 37]. As such, our study confirms the findings of others, i.e. an increased risk of unintended pregnancy among women who suffer(ed) IPV.

Furthermore, our study showed that women with a history of childhood abuse (<18 years of age) are at an increased risk of an unintended pregnancy. The vast body of research on childhood abuse shows that victims are more likely to achieve less education, have more physical and psychological complaints and psychiatric and medical diagnoses, and partake in harmful behaviour such as substance abuse. All of which may influence fertility control. In addition is re-victimization common, i.e. women abused in childhood become victims of adult abuse [36].

While we found significant associations for all the types of abuse for the total sample, the same was not true when we analysed by participating country. This is most likely due to the reduced statistical power of these smaller sub-samples.

A limitation of our study is that we have no information if the father of the child intended the pregnancy. This could have given us more information on the possible pathways of why women with a history of abuse more frequently have an unintended pregnancy. Studies suggest that pregnancy coercion, birth control sabotage and reproductive control by the partner are part of the mechanism which increases the risk for unintended pregnancy among women experiencing intimate partner violence [22, 23]. Neither have we information on the use of contraceptives. Women using contraceptives while becoming pregnant are more likely to have an unwanted pregnancy. Studies suggest that the negative impact of unintended pregnancies is greater for unwanted pregnancies than for mistimed pregnancies [16, 38].

We found significant differences in prevalence of unintended pregnancy between the countries participating in this study. In particular, Belgium and Denmark had a lower prevalence than Estonia, Norway and Sweden while Iceland had a higher prevalence. This could be related to the fertility rate in the respective countries. The thought behind this being that the more children being born per woman the more likely they are to be unplanned. Belgium and Denmark had a lower fertility rate than Norway, Sweden and Iceland in 2008–2010 [39]. However, Estonia had the lowest fertility rate in 2010 among the countries participating in our study and yet their prevalence of unintended pregnancy was similar to Norway and Sweden. Research suggests that differences in the prevalence of unintended pregnancy is influenced by social, religious and cultural values which influence the use of contraceptives, termination of pregnancy, and how family constellations and children are valued [19, 20]. There are clear cultural, religious and possibly social differences between the participating countries. The benefits and sanctions women experience when becoming pregnant may also contribute to the differences. The extent of maternity leave and monetary compensation varies greatly between countries. For example, in Norway, a woman has 10–12 months of paid maternity leave after the birth, provided she has worked 6 of the past 10 months. In Belgium, maternity leave is limited to 10 weeks after birth. There are small differences between the countries when it comes to access and price of contraceptives.

Although we recruited women attending routine antenatal care, just a few of them may not have intended to continue with their pregnancy. We lack mode of delivery for 308 of the women who filled out the questionnaire. Among these, we found five women who reported an unplanned pregnancy and who had a gestational length of less than 17 weeks. All five were from Estonia and could still have decided to have a termination of pregnancy. However, the numbers are so few that they would not have affected our overall results.

The large sample of unselected pregnant women from six European countries is a major strength of this study. In addition is our study prospective, i.e. women reported pregnancy intention while pregnant. This is thought to give a more correct estimate of unintended pregnancies as studies suggest that there is a pronounced tendency for births which prospectively were classified as unwanted to be retrospectively described as wanted or mistimed [39].

We assessed the association of unintended pregnancy with physical, sexual and emotional abuse either as a child or as an adult. This is in contrast to other studies, which are mostly limited to intimate partner violence/domestic violence and none investigate the when they investigate the association between abuse and pregnancy intention, [11, 12, 21, 37]. A strength of our study is that abuse was measured using the 10 descriptive questions of the Norvold Abuse, which has been validated among Swedish women attending gynaecology outpatients departments and has been previously used in several of the Scandinavian countries also included in our present study [29, 30]. The population in these studies was similar to the population in our study. However, a limitation of the study is that the questions have not been validated in other countries beside Sweden [30].

It is difficult to compare the prevalence of intended pregnancy in our study with studies using the terms mistimed and unwanted pregnancy [15]. Our findings are in agreement with a study from Southern Sweden who reported 14.1 % of unintended pregnancies among those without and 23.1 % among those with a history of abuse [14].

## Conclusion

Our findings suggest that women, who experienced physical, emotional or sexual abuse in childhood, adulthood or both, are more likely to experience an unintended pregnancy. The strongest association was found for abuse experienced within the past 12 months. If, as we supposed, women experienced this recent abuse from their current partner/father of the unborn child, they may be concerned for the welfare of their child as well as their own and the consequences of having a baby together with this person may have. From all perspectives, these are vulnerable women, who should be identified and offered appropriate services during pregnancy.

## Additional files

Additional file 1: Table S1. Crude and adjusted association for unintended pregnancy by country of participation, the Bidens study, N = 7102. Additional file 2: Table S2. Crude and adjusted OR for unintended pregnancy by a history of abuse at a mild, moderate or severe level, the Bidens study, N = 7102. Additional file 3: Figure S1. The adjusted Odds Ratios for unintended pregnancy and any lifetime abuse by participating country, the Bidens study, N = 7102.
