# Supplementary material for: Pregnancy intendedness and the association with physical, sexual and emotional abuse – a European multi-country cross-sectional study
Source: BMC Pregnancy Childbirth. 2015 May 26;15:120. doi: 10.1186/s12884-015-0558-4 (PMC4494794; doi:10.1186/s12884-015-0558-4)
Supplement: Additional file 1: Table S1. — Crude and adjusted association for unintended pregnancy by country of participation, the Bidens study, N = 7102. [file 12884_2015_558_MOESM1_ESM.docx]

|  | Crude OR | Adjusted OR^§^ |
| --- | --- | --- |
| Belgium | Ref | Ref |
| Iceland | 3.18 (2.39–4.25) | 3.15 (2.34–4.44) |
| Denmark | 1.34 (1.01–1.77) | 1.71 (1.25–2.35) |
| Estonia | 2.67 (2.04–3.50) | 2.05 (1.51–2.79) |
| Norway | 2.41 (1.88–3.07) | 2.63 (1.99–3.47) |
| Sweden | 2.73 (2.09–3.56) | 3.11 (2.26–4.30) |

Supplementary Table 1. Crude and adjusted association for unintended pregnancy by country of participation, the Bidens study, N=7102

^§^controlled for age, education, occupation, economic hardship and gestational age.
