# Supplementary material for: Pregnancy intendedness and the association with physical, sexual and emotional abuse – a European multi-country cross-sectional study
Source: BMC Pregnancy Childbirth. 2015 May 26;15:120. doi: 10.1186/s12884-015-0558-4 (PMC4494794; doi:10.1186/s12884-015-0558-4)
Supplement: Additional file 2: Table S2. — Crude and adjusted OR for unintended pregnancy by a history of abuse at a mild, moderate or severe level, the Bidens study, N = 7102. [file 12884_2015_558_MOESM2_ESM.docx]

Supplementary Table 2. Crude and adjusted OR for unintended pregnancy by a history of abuse at a mild, moderate or severe level, the Bidens study, N=7102

|  | n | Crude OR | Adjusted OR^§^ |
| --- | --- | --- | --- |
| Not any abuse |  | 1 | 1 |
| **Emotional abuse** |  |  |  |
| Mild | 1088 | 2.08 (1.78–2.43) | 1.60 (1.03–2.62) |
| Moderate | 150 | 2.29 (1.59–3.27) | 1.58 (1.07–2.33) |
| Severe | 106 | 2.01 (1.30–3.10) | 1.64 (1.03–2.62) |
| **Physical abuse** |  |  |  |
| Mild | 1021 | 1.98 (1.68–2.32) | 1.45 (1.22–1.73) |
| Moderate | 571 | 1.99 (1.62–2.43) | 1.52 (1.23–1.89) |
| Severe | 161 | 1.71 (1.18–2.47) | 1.64 (1.11–2.42) |
| **Sexual abuse** |  |  |  |
| Mild | 799 | 2.07 (1.73–2.46) | 1.69 (1.40–2.04) |
| Moderate | 241 | 2.10 (1.57–2.81) | 1.72 (1.25–2.35) |
| Severe | 76 | 2.17 (1.32–3.59) | 1.61 (0.93–2.77) |

^§^controlled for age, education, occupation, economic hardship and gestational age.
