# Supplementary material for: Pregnancy intendedness and the association with physical, sexual and emotional abuse – a European multi-country cross-sectional study
Source: BMC Pregnancy Childbirth. 2015 May 26;15:120. doi: 10.1186/s12884-015-0558-4 (PMC4494794; doi:10.1186/s12884-015-0558-4)

Supplementary Figure 1. The adjusted Odds Ratios for unintended pregnancy and any lifetime abuse by participating country, the Bidens study, N=7102


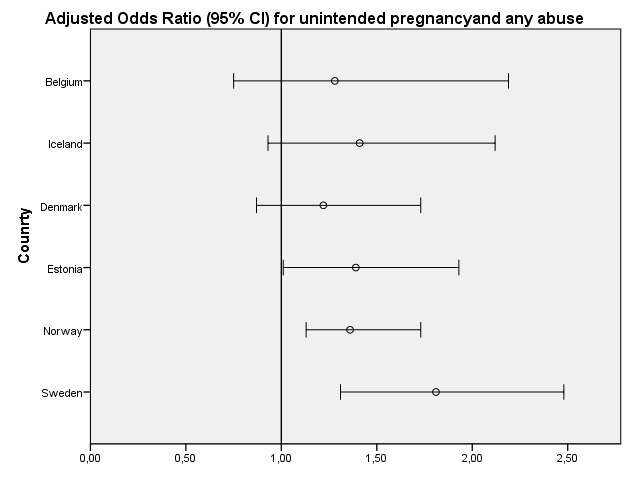

Supplement: Additional file 3: Figure S1. — The adjusted Odds Ratios for unintended pregnancy and any lifetime abuse by participating country, the Bidens study, N = 7102. [file 12884_2015_558_MOESM3_ESM.docx]
